# How much can we trust GPS wildlife tracking? An assessment in semi-free-ranging Crested Ibis *Nipponia nippon*

**DOI:** 10.7717/peerj.5320

**Published:** 2018-07-24

**Authors:** Dongping Liu, Lixia Chen, Yihua Wang, Jun Lu, Songlin Huang

**Affiliations:** Key Laboratory of Forest Protection of State Forestry Administration, Research Institute of Forest Ecology and Environment Protection, Chinese Academy of Forestry, Beijing, China

**Keywords:** GPS tracking, HDOP, Crested ibis, Location accuracy, PDOP, Positioning error, Acclimation

## Abstract

GPS tracking has been increasingly used for wildlife studies in recent decades, but its performance has not been fully assessed, especially for newly developed lightweight transmitters. We assessed the performance of eight GPS transmitters developed in China by attaching them to Crested Ibises *Nipponia nippon* confined to two acclimation cages mimicking real habitats. We calculated the distance between GPS locations and the centroid of the cages as the positioning error, and used the 95% (95th percentile) positioning errors to define the accuracy. The positioning success averaged 92.0%, which is much higher than that of previous studies. Locations were not evenly distributed by Location Class (LC), with the LC A and B locations accounting for 88.7%. The observed 95% positioning error in the locations of LC A (9–39 m) and B (11–41 m) was quite accurate, while up to 6.9–8.8% of poor-quality locations were detected in LC C and D with >100 m or even >1, 000 m positioning error. Positioning success and accuracy were different between the test sites, probably due to the difference in vegetation structure. Thus, we argue that the tested transmitters could provide a large proportion of high-quality data for fine-scale studies, and a number of poor-quality locations that need attention. We suggest that the HPOD (horizontal dilution of precision) or PDOP (positional dilution of precision) be reported instead of the LC as a measurement of location accuracy for each location to ensure identification and filtering of implausible locations.

## Introduction

GPS tracking has been increasingly used for wildlife studies in recent decades, which has revolutionized our understanding of individual wildlife movement, behaviour and spatial ecology (Rodgers & Anson, 1994; Cagnacci et al., 2010; Wilmers et al., 2015). Compared to Argos satellite tracking, GPS tracking can provide abundant high-quality data on animal movement, enabling fine-scale studies and conservation even for species in extreme environments with poor accessibility (Block et al., 2011; Hawkes et al., 2013; Ramírez-Macías et al., 2017).

Despite the high quality of tracking data, a number of factors may influence GPS location accuracy and positioning success (ratio between the observed and expected number of locations), including the effects of the terrestrial atmosphere (influencing the satellite signal), satellite constellation (influencing the number of satellites contacted), environment of the transmitters (habitat, topography, and weather), and behaviour pattern and movement intensity of the tagged animal (Trimble Navigation Limited, 1998; Cargnelutti et al., 2007; Recio et al., 2011; Mattisson et al., 2016; Byrne et al., 2017). Therefore, it is crucial to assess the accuracy of GPS tracking to understand the quality of location data and how the environment and animal behaviour influence the data quality, so that the data can be exploited properly and data correction can be processed for fine-scale studies such as those of animal movements in relation to landscape (Rempel & Rodgers, 1997). Rigorous testing of positioning accuracy is an essential prerequisite, especially for newly developed GPS devices.

Tests of GPS transmitter performance have been carried out over the last two decades, mainly on large mammals due to the weight and size limitations of GPS devices (Edenius, 1997; Cargnelutti et al., 2007; Yamazaki et al., 2009; Schwartz et al., 2017). Due to the improvements in microelectronics and battery technology, lightweight GPS transmitters have been developed in recent years that make it possible to track a variety of small-sized animals (Freeman et al., 2009; Pfeiffer & Meyburg, 2015; Liu et al., 2017). Lightweight GPS transmitters are usually deployed close to the ground where they are subjected to complex micro-topographical features, likely resulting in different performance from their larger counterparts, which has not been fully assessed (Recio et al., 2011).

In recent years, a series of lightweight GPS transmitters have been developed in China to meet the need for wildlife tracking. Compared to their mainstream counterparts, these transmitters are much more affordable for researchers, and thus are in increasing need especially in developing countries. However, the performances of these transmitters have not been exploited. In this study, we provide the first performance test of eight lightweight GPS transmitters newly developed in China by attaching them to semi-free-ranging Crested Ibises *Nipponia nippon*. As part of a re-introduction project, captive Crested Ibises were GPS tagged and confined to two large acclimation cages where they could acquire survival skills in conditions mimicking real habitats before they were released. The tracking data would help understand the movement and habitat selection of the Crested Ibises in the acclimation cage, and thus contribute to evaluate whether these birds could survive in the wild (IUCN, 1998). The objectives of this study were to detect: (1) the positioning success of the tested GPS transmitters; (2) the 95% (i.e., 95th percentile) horizontal positioning error for each Location Class; and (3) the proportion of poor-quality locations (with >100 m positioning error) in each Location Class. We also examined and discussed the influence of surrounding vegetation on transmitter performance by comparing the two test sites, and provided suggestions on data reporting improvement by the manufacturer and the proper use of GPS tracking data.

**Figure 1:**
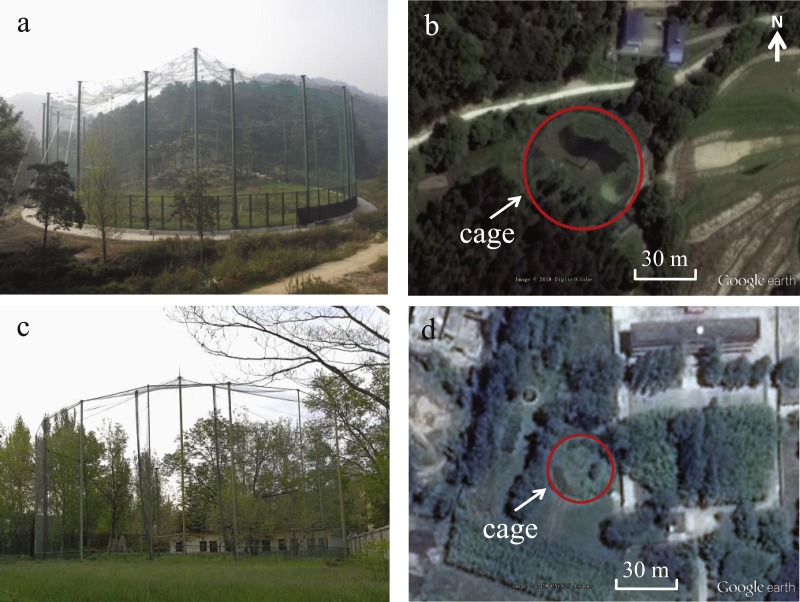
Photos and images showing the size and surroundings of the acclimation cages used as test sites at DZ (A and B) and TC (C and D). The acclimation cage at DZ was relatively large in size and open in surroundings compared to that at TC. Photos credit: Dongping Liu. Map data: Google Earth, DigitalGlobe.

## Methods

### Study sites

Tests were conducted in two acclimation cages for Crested Ibis situated at Dongzhai National Nature Reserve (hereafter, DZ) of Henan Province, China and Tongchuan (hereafter, TC) of Shaanxi Province, China (Fig. 1). Both of the cages are cylinder-shaped and made of nylon net (mesh size: 70 mm × 70 mm) held up by a number of central and peripheral columns, with the central column higher than the peripheral ones. The metal peripheral columns are approximately 10 m apart, and between each peripheral column and the central column, there is a steel rope to hold up the covering nylon net. Inside the acclimation cages, there are various habitats including trees, wetlands and grasslands that enable imitation of real wildlife monitoring situations. The acclimation cage at DZ (31.9681°N, 114.2795°E), located in an open valley 121 m above sea level, is characterized by being relatively open and having large space (30 m in radius and 20–32 m in height). Roost trees, dominated by *Metasequoia glyptostroboides*, occupy approximately 10% of the cage and are solely distributed in the southwestern part of the cage (Fig. 1). The remaining habitats are quite open and dominated by short grass and shallow water wetland. The acclimation cage at TC (35.0477°N, 108.8220°E), located at an altitude of 853 m, is surrounded by trees and buildings and relatively small in size (13 m in radius and 15–18 m in height). Roost trees (*Pinus massoniana* and *Diospyros kaki*) are evenly distributed in the cage. Therefore, transmitters tested at TC would be subjected to denser vegetation. In these acclimation cages, Crested Ibises can be free ranging, and take advantage of the mimicking real habitats and enough space to behave normally and acquire survival skills before release to the wild.

### GPS transmitters, attachment and tests

During 2014–2015, five 25-g GPS-GSM transmitters (model: HQBN2525; dimensions: 60 mm (length) × 30 mm (width) × 25 mm (height)) and three 27-g GPS-GSM transmitters (model: HQBN3527; dimensions: 60 mm (length) × 27 mm (width) × 35 mm (height)) manufactured by Hunan Global Messenger Technology Corporation, Limited, China (hereafter, HQXS) were tested at DZ and TC. All of the transmitters were solar-powered and programmed to position every hour. The tracking data from the transmitters were transmitted through the GSM system once a day. If GSM service was unavailable, data were stored onboard until GSM transmission was possible, with the ability to store up to 12,000 locations.

The transmitters were attached to Crested Ibises using a Teflon ribbon back harness. All the transmitters were glued with a cushion to ensure a total height of approximately 40 mm to reduce the masking of the solar panel by feathers. The total weight of the transmitter and harness was 1.6–1.9% of an ibis’s body mass. Birds were released into acclimation cages immediately after processing. All birds were confined to acclimation cages during the test period until they were released into the wild.

### Data processing and statistical analysis

The manufacturer provided a set of information for each successful location, including date, time, coordinates, speed, locomotion direction, altitude, temperature, voltage, and Location Class. Four Location Classes (hereafter, LC) from A to D with an advertised accuracy of 5 m, 10 m, 20 m and 100 m, respectively, computed from HDOP (horizontal dilution of precision, an indication of the possible horizontal accuracy of a location) or PDOP (positional dilution of precision, an indication of the possible accuracy of a location) at a probability of 95%, were reported by the manufacturer as a measurement of location accuracy (i.e., horizontal positioning error) (HQXS, 2017).

We used the central column of the acclimation cage as the reference point, measured by the average of 30 position fixes collected with a handheld Garmin GPSmap 60CSx. We plotted all of the locations from transmitters in ArcView GIS (version 3.3; Environmental Systems Research Institute, Redlands, California) and used the XTools extension to compute the distance between each GPS location and the reference point as the location error. Since the advertised location accuracy was estimated at a probability of 95%, we calculated the 95th percentiles from the ranked location errors (i.e., the horizontal positioning error at a 95% probability) for each LC to define the positioning accuracy. Because the tagged birds were free-ranging in the acclimation cage, the use of the central column of the cage as the reference point might incur a positive measurement error equals to the distance between the birds and the central column of the cage with a maximum of the radius of cage. This measurement error will result in an overestimate of the location error. To address this bias, we reported the positioning accuracy in a range, with the lower limiting value as the 95% location error minus the radius of the cage.

We used a Chi-square test to compare differences in positioning success among transmitters and differences in the proportion of locations in each LC among transmitters. We checked the data for normality, and used a Mann–Whitney test to detect differences in positioning success between DZ and TC. We used a Kruskal Wallis test to compare differences in the positioning error among locations of the four LCs. All statistical analyses were performed using the SPSS statistical software package (version 22.0, IBM 2013).

### Ethical note

All data collected as part of this study were approved by the National Bird Banding Center of China (No. NBBC20140210). This test was conducted as part of a re-introduction project for the Crested Ibis, under the supervision of a team of experienced ornithologists. The conditions in the cages and the acclimation procedure were in line with the IUCN Re-introduction Guidelines.

## Results

The complete dataset for the eight transmitters contained 4084 locations, with an average positioning success of 92.0% (Table 1). Positioning success differed significantly among the eight transmitters (Chi-square test, *χ*^2^ = 19.073, *df* = 7, *P* = 0.008) and was higher at DZ than at TC (Mann–Whitney test, *Z* =  − 36.862, *P* < 0.001). The proportion of locations in each LC differed among the eight transmitters (Chi-square test, *χ*^2^ = 1278.955, *df* = 21, *P* < 0.001). On average, the LC B locations accounted for up to 67.5%, while the LC D locations constituted the smallest proportion of 1.4% (Table 1).

**Table 1:** Positioning success and location distribution by Location Class of eight GPS transmitters tested at two acclimation cages at DZ and TC. The GPS transmitters were programmed to position every hour. Four Location Classes, from A to D with advertised accuracy of 5 m, 10 m, 20 m and 100 m were reported as a measurement of location accuracy.

Series No.	Test site	Test period	Total locations (Positioning success)	No. locations by location class			
				A	B	C	D
1	DZ	Jul 18—Aug 11, 2014	486 (81.0%)	19	443	17	7
2	DZ	Jul 18—Aug 11, 2014	594 (99.0%)	218	313	55	8
3	DZ	Jul 18–Aug 11, 2014	594 (99.0%)	15	505	63	11
4	DZ	Jul 18–Aug 11, 2014	528 (88.0%)	23	432	57	16
5	DZ	Jul 18–Aug 11, 2014	560 (93.3%)	21	467	67	5
6	TC	Mar 18–Mar 30, 2015	221 (70.8%)	33	127	56	5
7	TC	Mar 18–Apr 10, 2015	567 (98.4%)	358	195	13	1
8	TC	Mar 18–Apr 9, 2015	534 (96.7%)	177	273	79	5
Total			4,084	864 (21.2%)	2,755 (67.5%)	407 (10.0%)	58 (1.4%)

**Figure 2:**
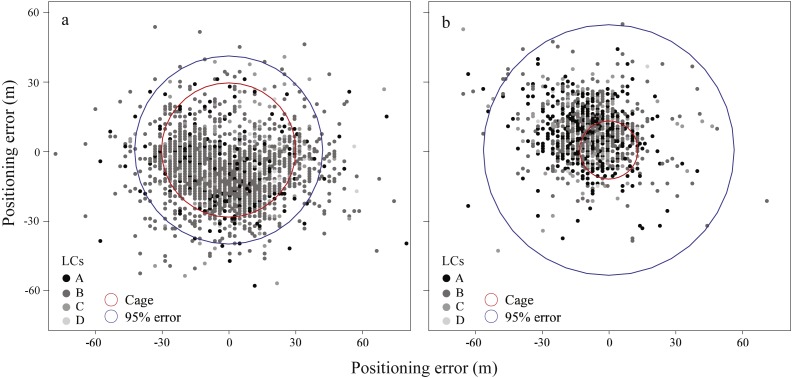
Map showing the location distribution relative to cage area and 95% positioning error bound at DZ (A) and TC (B). Locations were showed in graduated color for different Location Classes (LCs). Note that some extreme outliers were not showed in the map.

**Figure 3:**
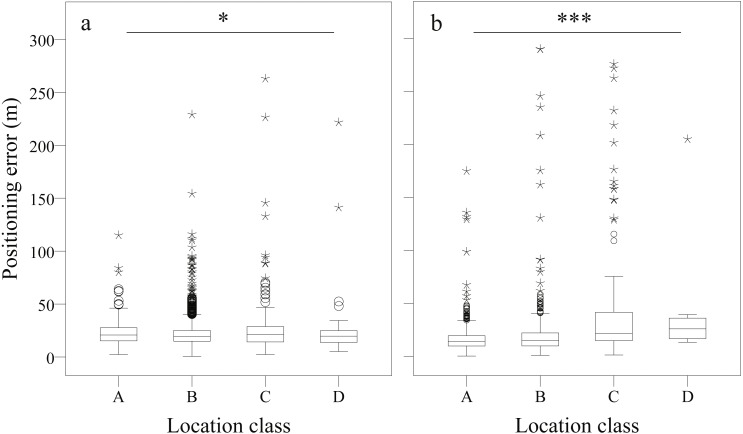
Boxplots showing the differences among positioning errors by Location Class at DZ (A) and TC (B). The open dots indicate mild outliers (Q3 + 1 IQR ∼ Q3 + 3 IQR), and the stars indicate extreme outliers (>*Q*3 + 3 IQR). Extreme Outliers with positioning error >300 m were not showed. * *P* < 0.05, *** *P* < 0.001. Significance levels were based on Kruskal Wallis test.

The distribution of locations relative to the test cages and bound 95% positioning error at DZ and TC is shown in Fig. 2. Significant differences were detected in the positioning error among locations of the four LCs at both DZ (Kruskal Wallis test, *χ*^2^ = 7.858, *df* = 3, *P* = 0.049) and TC (*χ*^2^ = 65.646, *df* = 3, *P* < 0.001) (Fig. 3). For the pooled data, the 95% positioning error in the locations of LC A, B, C and D was 39 m, 41 m, 230 m and 206 m, respectively (Table 2). Therefore, considering the measurement error caused by the size of the cage, the positioning accuracy of LC A, B, C and D would be 9–39 m, 11–41 m, 200–230 m and 176–206 m, respectively. Despite the high-quality of most locations, there were up to 1.6% of locations with >100 m errors. The proportion of these poor-quality locations was much higher for LC C (8.8%) and D (6.9%) compared to LC A (0.6%) and B (0.8%) (Chi-square test, *χ*^2^ = 145.162, *df* = 3, *P* < 0.001; Table 2). We also observed a higher proportion of poor-quality locations at TC (3.6%) than at DZ (0.7%)(*χ*^2^ = 45.978, *df* = 1, *P* < 0.001).

**Table 2:** The median and 95% positioning errors for tested transmitters at DZ and TC, and for the pooled data. The percentage of locations with >100 m location error, defined as low-quality locations, was also reported.

Test site	Location class	*N*	Median horizontal error (m)	95% horizontal error (m)	Range (m)	Percentage of locations >100 m error
DZ	A	296	21	43	2–115	0.3%
	B	2,160	19	41	1–1,539	0.5%
	C	259	21	69	2–1,929	2.3%
	D	47	20	105	5–222	4.3%
	**All**	**2,762**	**20**	**42**	**1–1,929**	**0.7%**
TC	A	568	15	36	1–175	0.7%
	B	595	16	48	1–4,494	2.0%
	C	148	22	507	2–1,532	20.3%
	D	11	26	—	14–450	18.2%
	**All**	**1,322**	**16**	**56**	**1–4,494**	**3.6%**
DZ + TC	A	864	17	39	1–175	0.6%
	B	2,755	19	41	1–4,494	0.8%
	C	407	22	230	2–1,929	8.8%
	D	58	20	206	5–450	6.9%
	**All**	**4,084**	**19**	**44**	**1–4,494**	**1.6%**

## Discussion

A series of lightweight GPS transmitters have been newly developed in China in recent years to meet the increasing need for wildlife tracking. To the best of our knowledge, our study presented the first performance assessment of GPS transmitters from HQXS, one of the major brands in China, which will provide an essential prerequisite for the application of these transmitters and contribute to the proper use of GPS wildlife tracking data. Since animal behaviours such as locomotion, hiding and diving may influence GPS location accuracy and positioning success (Recio et al., 2011; Mattisson et al., 2016), it is more reasonable to assess transmitter performance on free-ranging animals compared with using a stationary test. The acclimation cages provided ideal test sites for transmitter performance on semi-free-ranging Crested Ibises in conditions mimicking real habitats, which allowed animals to behave more naturally compared with those in tests on controlled animals moving in selected routes (Cargnelutti et al., 2007; Forin-Wiart et al., 2015; De Weerd et al., 2015). However, our results should be interpreted cautiously because the behaviour pattern of the tagged Crested Ibises in the acclimation cage might differ from that of their wild counterparts and consequently affect the positioning accuracy. For instance, the birds would fly relatively less, and be subjected to denser vegetation due to the less open foraging wetlands in the cage. Depending on the frequency and strategy of food supplement, the time budget on foraging and roosting of the Crested Ibises in the cages might also differ significantly from that in the wild (Liu et al., 2008). In addition, because the tagged birds were free-ranging in the acclimation cages, the use of central column of the cages as the reference point might incur an estimate error for the positioning accuracy. To take this into account, we reported the positioning accuracy in a range to provided the basic prerequisite for the proper use of the tracking data.

We detected an average positioning success of 92.0%, which was much higher than that of GPS transmitters deployed on animals (69.3%) and similar to that in stationary tests (94.8%) based on a review of 35 journal articles (Cain et al., 2005). This indicated quite good positioning sensitivity of our tested transmitters. The observed 95% positioning error of the LC A and B locations was estimated to be 9–39 m and 11–41 m, respectively, which was in line with the advertised accuracy (5 m for LC A and 10 m for LC B). The locations in these two LCs constituted 88.7% of the total locations, guaranteeing quite high quality of the tracking data overall. For the LC C and D locations, however, the observed positioning error (200–230 m for LC C and 176–206 m for LC D) was much higher than the advertised accuracy (20 m and 100 m, respectively). Moreover, 6.9–8.8% of the LC C and D locations were of poor-quality with >100 m or even >1,000 m positioning errors. Therefore, although most of the locations are supposed to be quite accurate, we should bear in mind that GPS tracking data may also include a few implausible locations and will need to be properly processed before use. Especially for fine-scale studies such as those of animal movements in relation to landscape, ornithologists are suggested to present the expected location error and its probability, and to make sure that the resolution of habitat data is not greater than the expected location error (Byrne et al., 2017).

Location accuracy is calculated by HDOP or PDOP and classified as four LCs for our tested transmitters (HQXS, 2017). This classification causes considerable information loss, and consequently, we cannot determine the exact accuracy of each location. We suggest the manufacturer reports the HDOP or the PDOP instead of the LCs for each location, so that we can identify and filter implausible locations for fine-scale studies. To compromise between the quantity and quality of the retained data, a HDOP value of 5 or a PDOP value of 10 was suggested as a threshold for gross data (Dussault et al., 2001; D’Eon & Delparte, 2005).

Our results revealed that there was lower positioning success and relatively more poor-quality locations at TC than at DZ, probably due to the relatively smaller cage and dense surrounding trees at TC. This was similar with many previous studies (Cargnelutti et al., 2007; Frair et al., 2010; Recio et al., 2011). Recio et al. (2011) demonstrated that vegetation structure played a critical role in the performance of GPS transmitters, i.e., low vegetation could only incur negligible impact, while dense forest might result in significant influence. This has an important implication in the appropriate use of GPS tracking data. Take Crested Ibises as an example; their daytime locations could be quite accurate since the species usually feeds in open areas with low vegetation. The night-time locations, however, might be subject to roost trees and will need to be carefully filtered for appropriate use.

## Conclusions

In conclusion, our study provided the first performance assessment of lightweight transmitters newly developed by HQXS in China, which could provide a large proportion of high-quality data. However, a number of factors, including vegetation structure, might have impacts on the positioning success and accuracy of these GPS transmitters, and result in a number of poor-quality locations. Thus, the tracking data need to be exploited properly and corrected carefully for fine-scale studies. We suggest the HPOD or the PDOP be reported instead of the LC as a measurement of location accuracy for each location to ensure the identification and filtering of implausible locations.

##  Supplemental Information

10.7717/peerj.5320/supp-1 Data S1 Location details derived from tested PTTs at DZ

10.7717/peerj.5320/supp-2 Data S2 Location details derived from tested PTTs at TC

## Additional Information and Declarations

### Data Availability

The following information was supplied regarding data availability: The raw data are provided in the Supplemental Files.
